# Quantification of myocardium at risk in ST- elevation myocardial infarction: a comparison of contrast-enhanced steady-state free precession cine cardiovascular magnetic resonance with coronary angiographic jeopardy scores

**DOI:** 10.1186/s12968-017-0359-1

**Published:** 2017-07-27

**Authors:** Rodney De Palma, Peder Sörensson, Dinos Verouhis, John Pernow, Nawzad Saleh

**Affiliations:** Karolinska Institutet, Department of Medicine, Unit of Cardiology, Karolinska University Hospital, Stockholm, Sweden

**Keywords:** Myocardium, myocardial salvage, cardiovascular magnetic resonance imaging

## Abstract

**Background:**

Clinical outcome following acute myocardial infarction is predicted by final infarct size evaluated in relation to left ventricular myocardium at risk (MaR). Contrast-enhanced steady-state free precession (CE-SSFP) cardiovascular magnetic resonance imaging (CMR) is not widely used for assessing MaR. Evidence of its utility compared to traditional assessment methods and as a surrogate for clinical outcome is needed.

**Methods:**

Retrospective analysis within a study evaluating post-conditioning during ST elevation myocardial infarction (STEMI) treated with coronary intervention (*n* = 78). CE-SSFP post-infarction was compared with angiographic jeopardy methods. Differences and variability between CMR and angiographic methods using Bland-Altman analyses were evaluated. Clinical outcomes were compared to MaR and extent of infarction.

**Results:**

MaR showed correlation between CE-SSFP, and both BARI and APPROACH scores of 0.83 (*p* < 0.0001) and 0.84 (*p* < 0.0001) respectively. Bias between CE-SSFP and BARI was 1.1% (agreement limits -11.4 to +9.1). Bias between CE-SSFP and APPROACH was 1.2% (agreement limits -13 to +10.5). Inter-observer variability for the BARI score was 0.56 ± 2.9; 0.42 ± 2.1 for the APPROACH score; -1.4 ± 3.1% for CE-SSFP. Intra-observer variability was 0.15 ± 1.85 for the BARI score; for the APPROACH score 0.19 ± 1.6; and for CE-SSFP -0.58 ± 2.9%.

**Conclusion:**

Quantification of MaR with CE-SSFP imaging following STEMI shows high correlation and low bias compared with angiographic scoring and supports its use as a reliable and practical method to determine myocardial salvage in this patient population.

**Trial registration:**

Clinical trial registration information for the parent clinical trial:

Karolinska Clinical Trial Registration (2008)

Unique identifier: CT20080014. Registered 04^th^ January 2008

**Electronic supplementary material:**

The online version of this article (doi:10.1186/s12968-017-0359-1) contains supplementary material, which is available to authorized users.

## Background

The clinical outcome following acute ST-segment elevation myocardial infarction (STEMI) is predicted by the final infarct size [1, 2]. This in turn is determined by a complex interplay between several factors including the duration of myocardial ischemia, the presence of collateral blood supply, reperfusion injury and left ventricular myocardium at risk (MaR). Therapies to restore myocardial perfusion and/or limit reperfusion injury, whether mechanical or pharmacological, are incompletely assessed by final absolute infarct size alone [3]. Evaluation of infarct size has to be performed in relation to the MaR. The myocardial salvage index, calculated as (MaR-final infarct size)/MaR, is a more robust surrogate for therapeutic efficacy. The reference technique for assessing the MaR is nuclear single photon emission computed tomography (SPECT) [4–6]. This imaging modality, however, is not practical in the clinical setting as it requires acute imaging when a patient may remain clinically unstable as well as two subsequent image acquisitions. Furthermore, it has relatively limited spatial resolution and involves exposure to ionizing radiation. It is therefore of interest that several studies have demonstrated that cardiovascular magnetic resonance imaging (CMR) is equivalent to SPECT at 7 days following STEMI for assessing MaR using contrast-enhanced steady-state free precession cardiac magnetic resonance (CE-SSFP), or T2-weighted sequences in a single examination [4, 7–9].

CE-SSFP is not yet widely used for the assessment of MaR but has been shown to be more robust than T2-weighted imaging in multi-center, multi-vendor studies [10, 11]. Given the potential advantage of combining CE-CMR with SSFP and LGE to evaluate both MaR and final infarct size in this setting further evidence of its utility as a surrogate for clinical outcome is needed. Angiographic assessment of myocardium at risk has been shown to predict clinical outcome following myocardial infarction [12, 13]. A comparison of CE-SSFP and such angiographic evaluation has not been undertaken previously.

### Aim

The aim of the current investigation was therefore to evaluate the relationship between CE-SSFP and two validated angiographic scoring techniques for anatomical left ventricular myocardial area at risk: the BARI (Bypass Angioplasty Revascularization Investigation Myocardial Jeopardy index) score and the modified APPROACH (Alberta Provincial Project for Outcome Assessment in Coronary Heart Disease) score [12, 13]. Clinical outcome data were also evaluated in the context of MaR and extent of infarction.

The main hypothesis tested was that CE-SSFP provides a coherent determination of MaR, when compared with angiographic scoring techniques.

## Methods

Consecutive patients from a randomized trial evaluating post-conditioning during STEMI were analyzed retrospectively. This study was a single-center single-blinded prospective clinical trial evaluating the benefit of intracoronary balloon–mediated ischemic post-conditioning on myocardial infarct size in patients presenting with STEMI the setting of a primary percutaneous coronary intervention service with study recruitment between 1^st^ January 2009-31^st^ December 2009 and has been described in detail previously [14]. In brief, eligibility consisted of a presentation within 6 h compatible with acute ischemia, ST elevation on a 12 lead electrocardiogram (>0.1 mV in two contiguous leads, >0.2 mV in V1-V3, or new left bundle branch block and Thrombolysis in Myocardial Infarction (TIMI) 0 flow in an identifiable infarct-related artery on invasive coronary angiography. Exclusion criteria consisted of: previous myocardial infarction including the presence of pathological Q waves on the resting electrocardiogram, surgical revascularization, cardiogenic shock, a presentation with cardiac arrest, treatment with metformin, absolute contraindication to CMR, atrial fibrillation and known renal impairment (defined as creatinine >150 micromol/l).

### Invasive coronary angiography

Invasive coronary angiography was performed to confirm complete coronary occlusion in the infarct related artery and determine the BARI and Modified APPROACH scores. Percutaneous coronary intervention (PCI) was then performed according to local standard procedures at the discretion of the individual physician. The intervention was completed by a coronary angiographic acquisition to determine final TIMI grade flow.

Visual angiographic scoring, using the BARI and modified APPROACH systems, was carried out retrospectively using pre- and post-PCI images. Two experienced assessors, each with direct clinical experience of more than 2000 invasive procedures, and blinded to the CMR results scored the angiograms. The Rentrop scoring system was used to assess collateralization (Table 2).

### Post-conditioning protocol

After invasive coronary angiography, patients were randomized to primary PCI only or primary PCI followed by post-conditioning. This was performed by intracoronary occlusion using the PCI balloon to a pressure of 2-4 atmospheres for four cycles of 60 s starting 60 s after the initial reperfusion [14].

### Cardiovascular magnetic resonance imaging

A standard CMR was scheduled between 5-7 days after admission. These investigations were performed in the supine position with an eight-channel cardiac coil by means of a 1.5 Tesla system (Signa Excite TwinSpeed, General Electric Healthcare, Waukesha, Wisconsin, USA) during vector-ECG monitoring. Intravenous gadolinium-DTPA chelated contrast (0.2 mmol/kg; Omniscan, GE Healthcare) was administered before positioning the patient in the scanner. The image protocol included scout images, localization of the short axis and then covering the whole left ventricle (LV) with cine balanced steady-state free precession (SSFP) images retrospectively-gated. Baseline images were acquired 5 min after contrast administration. The following parameters were used; SSFP (echo time (TE) 1.58 milliseconds (ms), repetition time (TR) 3.61 ms, flip angle 60°, 25 phases, 8 millimetres (mm) slice, no inter-slice gap, matrix 226x226). Late gadolinium enhancement (LGE) images were acquired 15-20 min after contrast injection using an inversion recovery gradient echo sequence (TE 3.3 ms, TR 7.0 ms, inversion time 180-250 ms to null the myocardium, 8 mm slice, no inter-slice gap, matrix 256x192) and the same slice orientation as cine SSFP images [15, 16].

Cardiac triggering was set for diastole to reduce motion artifacts. Each slice was obtained during end-expiratory breath holding. Two-, three- and four-chamber views were also obtained to confirm the findings.

CMR images were analyzed off-line at a later session using freely available segmentation software (Segment V.1.8 R0857; http://segment.heiberg.se/) [17]. End-diastolic and end- systolic volumes, ejection fraction, stroke volume and left ventricular volume were calculated on cine SSFP sequences. Infarct size was quantified using an automated quantification method that has been validated ex vivo and in vivo in which account is made for partial volume effects [18, 19]. The extent of infarction is expressed both in absolute mass and as the percentage of MaR with LGE.

**Fig. 1:**
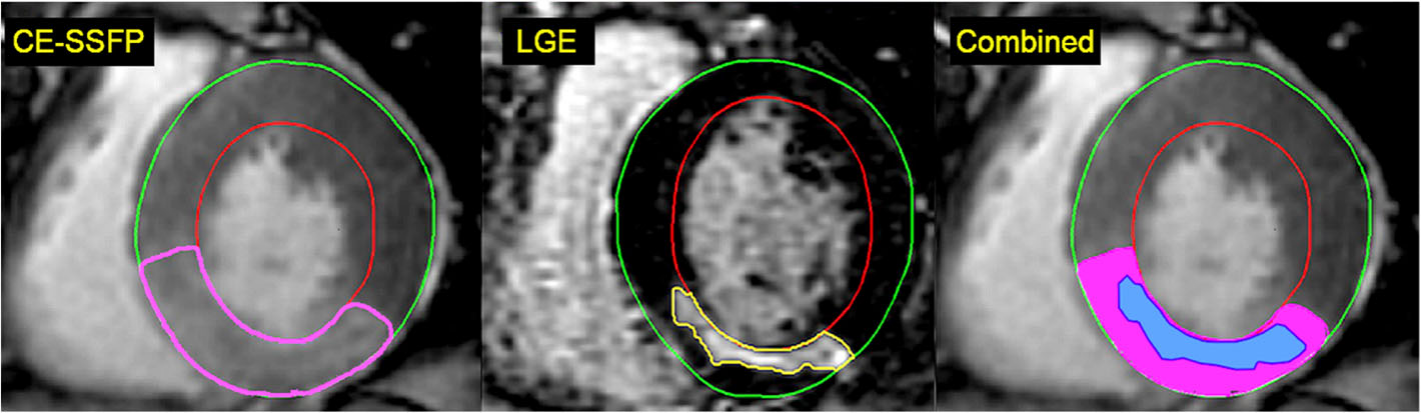
Example of MaR and myocardial salvage index using contrast-enhanced SSFP magnetic resonance imaging. Corresponding left ventricular short axis views from a patient with an inferior STEMI caused by a right coronary artery occlusion. The epicardium is delineated by a *green* border, and the endocardium by a *red* border. The myocardium at risk (MaR) in the inferior segment is determined by area of early gadolinium enhancement on SSFP (delineated by a *pink* border in the *panel to the left*). The size of infarction in the inferior segment is determined by the area of late gadolinium enhancement delineated by a *yellow* border in the *middle panel*. Automatic computer-aided superimposition of the infarcted area and the MaR area can be used to calculate the myocardial salvage index (as a ratio of the two areas, 1-scar area/MaR), *panel to the right*. MaR = myocardium at risk; CE-SSFP = contrast-enhanced steady-state free precession; STEMI = ST-elevation myocardial infarction; LGE = late gadolinium enhancement



MaR determined from CE-SSFP was assessed according to previously described methodology [9]. Endo- and epicardial borders were manually traced in all short-axis slices in both end-diastole and end-systole followed by manual delineation of the hyper-intense regions in end-diastole and end-systole, by one experienced observer (level 2 CMR certified and with 12 years of CMR experience) blinded to the LGE images and angiographic scores (Fig. 1). A second experienced observer (level 3 CMR certified and with 20 years of CMR experience) evaluated twelve patients for intra-observer variability. Hypo-intense myocardium within the area of increased signal intensity was regarded as microvascular obstruction and was included in the MaR. The values of MaR in end-diastole and end-systole were averaged and expressed as a percentage of LV mass, (Fig. 1 and Additional file 1: movie1).

**Additional file 1: Movie 1.** Contrast-enhanced SSFP cine loop of a full base-to-apex coverage short-axis slice set, with each slice moving through the full systolic and diastolic cycle, used to quantify MaR (corresponds to Fig. 1). (MOV 803 kb)


### BARI score

Left ventricular MaR was calculated by grading all terminating arteries. All branches were scored as 3, 2, 1 or 0 points, corresponding to, large, medium, small or absent respectively. The ventricular base to apex distance, approximated from the coronary angiogram, provided the basis for assessing the relative distribution of coronary branches. Branches were considered large if their length exceeded two- thirds of the distance from base to apex, medium if one-third to two-thirds the distance, small if less than one-third of the distance and absent if less than one-fifth of the distance from base to apex [12]. The risk score was calculated as a percentage of the left ventricular myocardial volume; by dividing summed scores of a jeopardized territory by the total score of the entire left ventricle.

### Modified APPROACH score

This score, which is based on pathological and necropsy studies [20, 21], is derived from a template that considers the culprit artery, dominance and lesion location (proximal or mid-vessel) together with the size (small, medium or large) of the remaining major non-culprit epicardial vessel [13]. The score in the template represents the percentage of LV MaR.

### Statistical methods

Data management and analyses were performed using EXCEL version 14.4.3 (Microsoft Corporation, Redmond, Wisconsin, USA) and GraphPad Prism V.5.00 (GraphPad Software, San Diego, California, USA).

Continuous data are presented as means with standard deviations (SD). Categorical data are presented as frequencies with percentages or medians with interquartile ranges (IQR).

The endpoint was the mean bias between the angiographic scores and MaR derived from the CE-SSFP images using Bland-Altman plots and expressed as mean and limits of agreement (2 x SD).

Pearson’s correlation coefficient was also used to evaluate the relationship between CE-SSFP and the angiographic risk scores.

Intra- and inter-observer variability was calculated as the SD of the difference between two calculations divided by the mean of the two observers and expressed as the mean+/- SD.

Statistical significance was accepted as *p* < 0.05.

## Results

A total of 89 patients out of a screened cohort of 795 presented with acute STEMI and TIMI 0 flow in the infarct-related artery and were eligible. Those not fulfilling the inclusion criteria were excluded prior to randomization (*n* = 706). Eleven patients were excluded following randomization due to poor CMR image quality, not fulfilling the post-conditioning protocol, or in-hospital death prior to imaging.

Those finally selected comprised 78 patients who underwent CMR examination at a median of 7 days (IQR 6-9) following admission.

Baseline clinical characteristics show the group to be young and predominantly male. Smoking and hypertension were present in a substantial proportion of patients but diabetes mellitus and previous symptoms of ischemic heart disease were infrequent.

The absence of prior symptomatic ischemic heart disease is also reflected by the small proportion of patients on cardiovascular preventive therapies prior to admission (Table 1).

**Table 1:** Clinical characteristics of the cohort

Variables	*N* = 78
On admission	
Age, years (SD)	62 ± 11
Male sex, n (%)	65 (83)
Body mass index, kg/m^2^ (IQR)	27 (25-30)
Ischemia time, minutes (IQR)	173 (140-239)
Current smoker, n (%)	24 (31)
Hypertension, n (%)	18 (23)
Dyslipidemia, n (%)	6 (8)
Previous angina, n (%)	9 (12)
Previous known diabetes mellitus, n (%)	2 (3)
Treatment on admission	
Aspirin, n (%)	5 (6)
Beta-blocker, n (%)	6 (8)
ACE/ARB, n (%)	7 (9)
Statin	6 (8)
Treatment at discharge	
Aspirin, n (%)	77 (99)
Clopidogrel, n (%)	78 (100)
Beta-blocker, n (%)	77 (99)
ACE/ARB, n (%)	44 (56)
Statin, n (%)	76 (97)

Data are presented as number of patients and percentage in brackets for dichotomous variables or median and interquartile range. *ACE* angiotensin converting enzyme inhibitor, *ARB* angiotensin receptor blocker, *IQR* inter-quartile range

In 35% of patients the culprit artery was the left anterior descending artery, in 57% of patients it was the right coronary artery, and in 8% it was the circumflex artery. No patients presented with a left main occlusion. Collateral circulation was visible in 18% (Table 2).

**Table 2:** Angiographic characteristics of the cohort

Variables	*N* = 78
Infarct related artery	
LAD	27 (35)
LCx	6 (8)
RCA	45 (57)
Disease pattern	
One-vessel disease	49 (63)
Two-vessel disease	22 (28)
Three-vessel disease	7 (9)
TIMI grade 3 flow after PCI	69 (88)
Collateral flow (Rentrop grade 0 or 1)	64 (82)
Collateral flow (Rentrop grade 2 or 3)	14 (18)

Data are presented as number of patients and percentage in brackets for dichotomous variables. *LAD* left anterior descending coronary artery, *RCA* right coronary artery, *LCx* left circumflex coronary artery, *TIMI* Thrombolysis In Myocardial Infarction, *PCI* percutaneous coronary intervention

Rentrop grade 0 = no filling of distal infarct vessel by collateral vessels

Rentrop grade 1 = filling of distal infarct vessel side branches only by collateral vessels

Rentrop grade 2 = partial filling of distal infarct main vessel by collateral vessels

Rentrop grade 3 = complete filling of distal infarct main vessel by collateral vessels

There were no significant differences in the percentage MaR based on culprit vessel or background coronary disease pattern between the methods (Table 3).

**Table 3:** Myocardium at risk by method used and angiographic characteristics

Variables	CMR	BARI	APPROACH
Total MaR	30 (7.7- 56.9)	28.5 (12-50)	28.0 (12-48)
Infarct related artery			
LAD	42 (32-49)	41 (33-46)	44 (30-47)
LCx	40 (35-46)	34 (32-37)	30 (28-40)
RCA	26 (22-32)	27 (22-29)	28 (24-28)
Disease pattern			
One-vessel disease	31 (25-44)	29 (27-40)	28 (27-44)
Two-vessel disease	31 (25-39)	30 (25-34)	28 (28-28)
Three-vessel disease	24 (22-32)	22 (22-26)	24 (22-28)
Collateral flow grade 0	31 (24-43)	30 (25-39)	28 (27-44)
Collateral flow grade 1	31 (27-39)	28 (27-36)	28 (27-29)
Collateral flow grade 2 or 3	21 (7-26)	27 (22-29)	28 (22-28)

Data on percentage myocardium at risk are presented as median and interquartile range (in brackets). There were no significant differences between the methods according to infarct-related artery or underlying coronary disease pattern. *LAD* left anterior descending coronary artery, *RCA* right coronary artery, *LCx* left circumflex coronary artery, *CMR* cardiac magnetic resonance, *MaR* myocardium at risk. There were no statistically significant differences between the MaR and collateralization for each of the angiographic techniques and CMR. Friedman test *p* = 0.157 and 0.06 respectively for collaterals 0 vs 2 and 3

All patients had a sufficient number and quality of angiographic projections to determine the culprit and non-culprit coronary anatomy.

### Relationship between CE-SSFP and angiographic scores

**Fig. 2:**
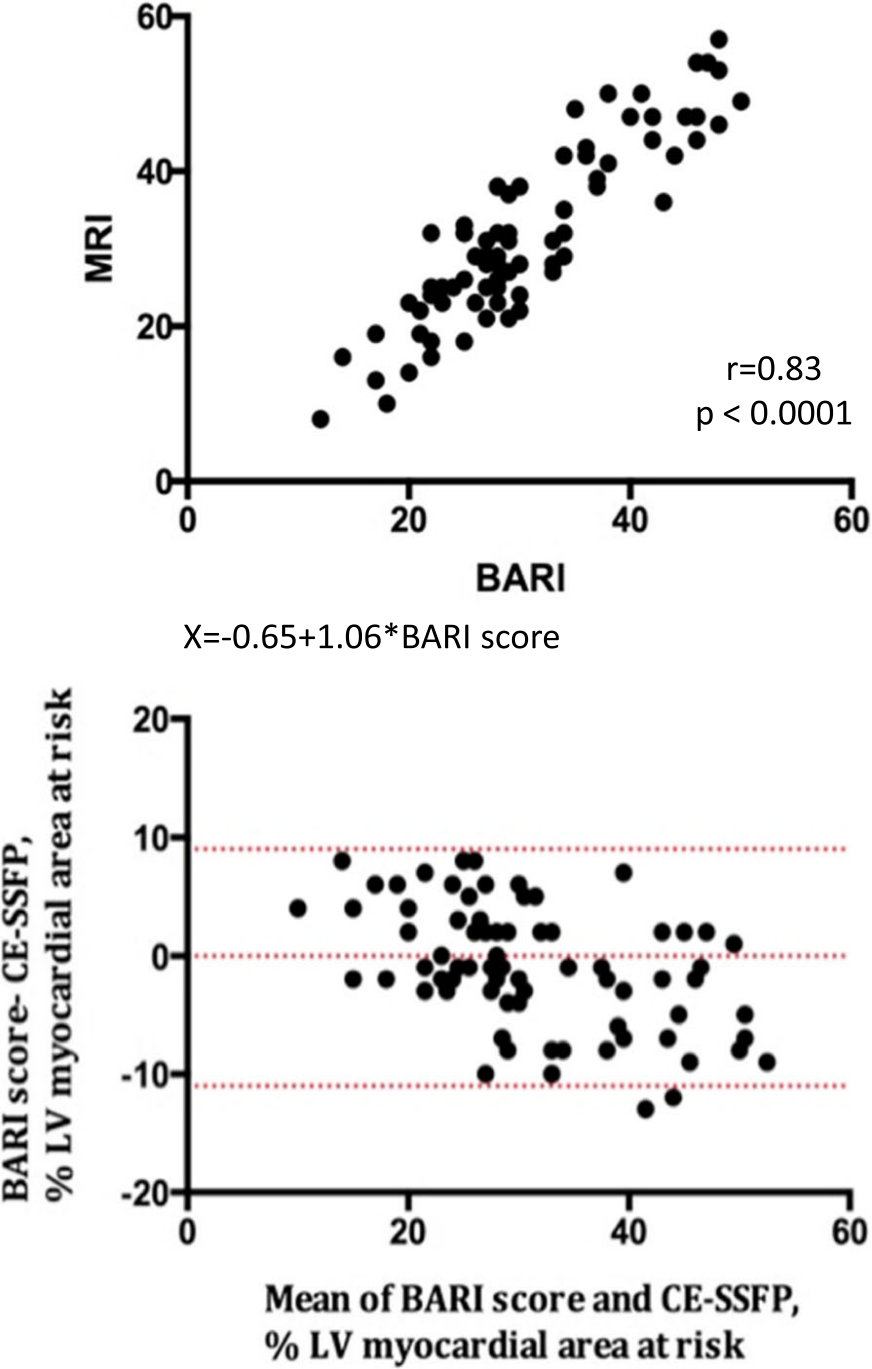
BARI score and myocardial area at risk measured with CE-SSFP. *Upper panel*: CE-SSFP shows a strong correlation with the BARI score (*r* = 0.83). *X* = -0.65 + 1.06*BARI score. *Lower panel*: Analysis of measurement variability between CE-SSFP and angiographic scores using Bland-Altman plots (difference in quantification of % area at risk versus mean of both methods). The mean difference was 1.1% between CE-SSFP and the BARI score. The *dotted lines* indicate limits of agreement from -11.4 to +9.1

**Fig. 3:**
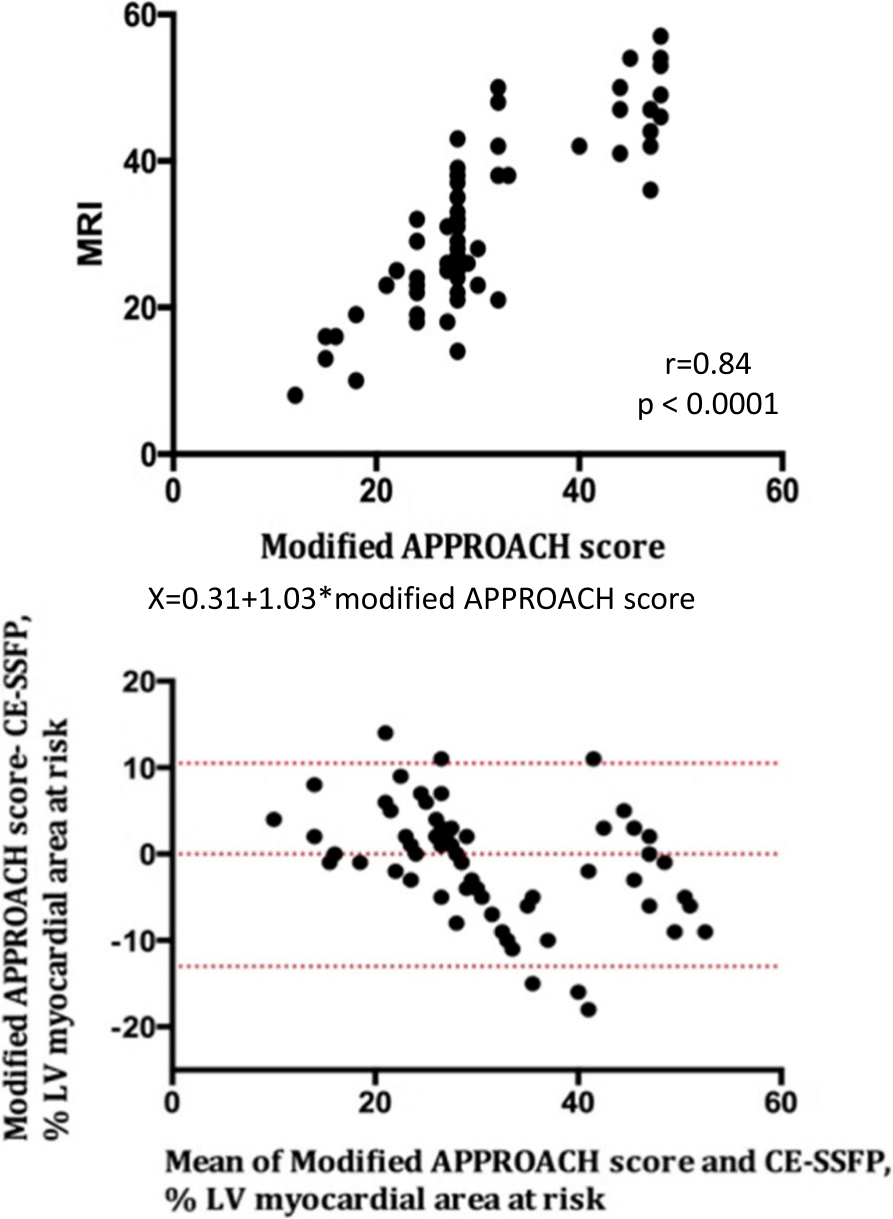
Modified APPROACH score and myocardial area at risk measured with CE-SSFP. *Upper panel*: CE-SSFP shows a strong correlation with the modified APPROACH score (*r* = 0.84). *X* = 0.31 + 1.03*modified APPROACH score. *Lower panel*: Analysis of measurement variability between CE-SSFP and angiographic scores using Bland-Altman plots (difference in quantification of % area at risk versus mean of both methods). The mean difference was 1.2% between CE-SSFP and the Modified APPROACH score. The *dotted lines* indicate limits of agreement from -13 to +10.5

There was a good linear correlation between the BARI and modified APPROACH scores and that derived from CMR (0.83 *p* < 0.0001 and 0.84 respectively, *p* < 0.0001) (Figs. 2 and 3). Bland-Altman analysis demonstrated that there was good agreement of the size of the MaR between CE-SSFP and angiographic scores (mean of 1.1%+/-5.2 with limits of agreement -11.4 to +9.1, and 1.2%+/-6.0 with limits of agreement -13 to +10.5, for the BARI and Modified APPROACH scores respectively) (Figs. 2 and 3).

### Reproducibility amongst observers

The angiographic and CMR estimated MaR did not differ between techniques (Table 3). The inter-observer variability showed a mean difference 0.56 ± 2.9% for the BARI score (*n* = 12, 95%CI = 0.84 to 0.28), 0.42 ± 2.1% for the Modified APPROACH score (*n* = 12, 95%CI = 0.70 to 0.14) and -1.4 ± 3.1% for the CE-SSFP technique (*n* = 12, 95%Ci = -0.36 to -2.44)). The intra-observer variability showed a mean difference 0.15 ± 1.85% for the BARI score (95%CI = 0.35 to -0.20), 0.19 ± 1.6% for the Modified APPROACH score (95%CI = 0.41 to -0.03) and for CE-SSFP -0.58 ± 2.9% (95%CI = -0.04 to -1.12)

## Discussion

The main finding is that contrast enhanced CE-SSFP CMR evaluation of MaR correlates well with angiographic jeopardy scores validated previously as surrogates for clinical outcome [22]. The bias was small with very mild overestimation, 1.1 and 1.2% for the BARI and Modified APPROACH scores respectively. The limits of agreement between the CMR and angiographic measurements were -11.4 to +9.1, and -13 to +10.5 for the BARI and Modified APPROACH scores respectively. There was consistent variability across the range of MaR measured.

Final infarct size in acute STEMI is a key determinant for clinical outcome and has to be determined in relation to MaR [1, 2]. Due to the large variability in absolute infarct size, very large study groups are needed to achieve adequate power in clinical studies having infarct size as an end-point. Myocardial salvage is a pragmatic surrogate measurement for therapeutic efficacy of interventions aiming at limiting infarct size since it normalizes the infarct size to MaR. Thus, the use of CMR and myocardial salvage index (rather than myocardial infarction size alone) substantially reduces the study group sizes needed to evaluate efficacy in clinical trials [23]. It is therefore of utmost importance to develop feasible methods that accurately determine MaR in the clinical setting. Furthermore, the evaluation of the peri-infarct zone, which is also a feature of CMR evaluation, can add incremental prognostic value [24].

### Cardiovascular magnetic resonance evaluation of myocardium at risk

CMR is increasingly recognized as equivalent to nuclear-based techniques for determining the MaR whilst also having inherent practical advantages. Several CMR sequences have been evaluated in this context including CE-SSFP [9, 11, 25, 26]. In 2012, Moral et al, compared BARI and APPROACH scores with T2-STIR and infarct endocardial surface area methods. They reported a good correlation between the different angiographic scores and their CMR-sequence [27]. The data presented here, as far as we are aware, represent the first comparison of CE-SSFP and angiographic jeopardy scores in the literature.

The advantage of this method is that it can be easily added to current standard clinical protocols, in scanners from all major vendors, simply by administering a single dose of gadolinium contrast agent before acquiring short-axis cine images. This shortens the CMR protocol in the scanner and the evaluation time after the examination since the same images can be used both for MaR determination and the evaluation of functional parameters. Unstable patients may also benefit from shorter CMR protocols.

Bright-blood T2-weighted sequences have been developed to increase the diagnostic accuracy of T2 CMR for depicting edema [28, 29]. One such sequence (ACUT2E) was more accurate for the determination of MaR and myocardial salvage than was dark-blood T2-weighted sequences, which results in underestimation of MaR [30]. Thus, CE-SSFP might be more accurate than dark-blood T2 since CE-SSFP showed larger MaR in a study by Ubachs et al [8]. Several limitations of different T2-weighted imaging protocols include a lack of consensus on the optimal quantification method, signal intensity variability within each slice and susceptibility to motion artefacts [7, 31, 32]. Newly developed T1- or T2-mapping techniques may improve accuracy considerably on these limitations when using motion correction and an absolute threshold for edema, [33–35] but further standardization and validation are still needed.

In patients, therefore, where there is no contraindication to gadolinium, the CE-SSFP may nevertheless still remain a more practical method to apply, particularly in time-constrained contemporary clinical working environments. Furthermore, the multi-phase acquisition of SSFP throughout the cardiac cycle allows for more robust delineation of endocardial and epicardial borders, less image artefacts, better spatial resolution, and as a consequence better inclusion rates of patients in clinical studies.

The mechanism for contrast-enhancement of the MaR during SSFP cannot be explained fully. The contrast in SSFP images is dependent on the sequence T2/T1 ratio. T2 relaxation times are based on the increase in tissue water content and mobility, which is a characteristic of MaR [36]. When using a gadolinium-based contrast agent, the T1 for the surrounding tissue is shortened. This is also the rationale for infarct visualization in T1-weighted inversion-recovery LGE imaging since the contrast agent distributes to the extracellular space. Here the concentration of an extracellular gadolinium-based contrast agent is increased due to an increased distribution volume in myocardium injured by ischemia [37–40]. It has been shown that even reversibly injured myocardium within the MaR has an increased distribution volume in the acute phase after an ischemic episode [41, 42]. The T2/T1 ratio in the entire MaR, including both reversible and irreversibly injured myocardium, is affected by the presence of gadolinium. This might therefore explain the increased signal intensity in the MaR seen by CE-SSFP.

There is an ongoing scientific discourse regarding the conditioning treatments of reperfusion injury and their effect on myocardial edema. Two recent studies [43, 44] have shown reduced MaR in the active group (local post-conditioning and remote pre-conditioning) of patients compared to controls, but the protocols and sequences used were not the same as the one used in our study. In addition, these studies used T2-STIR and T2-mapping respectively. The post-conditioning protocol in theory could have influenced the MaR calculated by CE-SSFP in the present study. The final infarct size and MaR, however, as judged by the extent of CE-SSFP and LGE was similar in both the control arm and post-conditioning arm. Furthermore, there were no differences in clinical outcomes between the groups. These observations suggest that any contribution of the treatment protocol to the MaR was minimal.

Collateral supply to an infarcted territory could also influence the final infarct size and MaR. Although there was no statistically significant difference between each of the angiographic quantification methods and the MaR for the extent of collateralization, there was a trend to significance with CE-SSFP evaluation (*p* = 0.157 and 0.06 respectively for Rentrop collaterals 0 vs a composite of Rentrop 2 and 3). This points to the intuitive consideration that CE-SSFP may have an added benefit as it takes the collateral supply into account whereas the rigid angiographic scoring techniques do not.

### Angiographic evaluation of myocardium at risk

The modified–APPROACH score is based on pathological and necropsy studies and is derived from a template that considers the culprit artery, dominance and lesion location (proximal or mid-vessel) together with the size (small, medium or large) of the remaining major non-culprit epicardial vessel. The score in the template represents the percentage of left ventricle at risk.

The BARI score bases the anatomical area at risk and the length and caliber of the epicardial coronaries and has been validated against post-mortem histological studies [21].

Both scores have been validated with respect to adverse clinical outcome in a large population-based cohort (20,067 patients) undergoing PCI with similar c-statistics for predicting one-year mortality, when added to simple baseline characteristics, of 0.85 and 0.84 for the APPROACH and BARI scores respectively [22]. For both these angiographic scores their validity is based predominantly on left anterior descending and right coronary artery infarctions. Challenges in the electrocardiographic diagnosis of circumflex occlusion leads to under-representation of this category of infarction [45].

The BARI score is more precise than the modified APPROACH score but is also more time-consuming. The modified APPROACH score on the other hand is subject to more observer variability. This study also reflects the broader variability of the latter compared to the former on Bland-Altman analysis. Whilst the BARI score takes into consideration stenotic lesions >50% and the Modified APPROACH score considers a lesion >70% as being significant, in the context of acute STEMI this difference in scoring is not manifest as the culprit vessel is occluded. The Modified APPROACH score is based on fixed assumptions of the myocardium at risk subtended by the location of a lesion in the coronary artery and hence clustering of high, intermediate and lower values can be seen when compared with CE-SSFP. There was no difference, however, in clinical outcomes and the absolute extent of infarction or in relation to MaR as estimated by either angiographic score or CE-SSFP. In addition, there was no difference in extent of infarction when adjusted for angiographically-evident collateralization. These data thus indicate that despite the precision of the BARI score, both angiographic techniques for estimating the size of MaR appeared to be clinically equivalent in this study.

Angiographic risk scores have been used to validate single-photon emission computed tomography as well as T2 weighted CMR [7, 46].

This study was not able to disprove the hypothesis that there was no difference between angiographic scoring and CE-SSFP for the assessment of MaR, hence validating its use for this purpose. The close correlation between the angiographic and CE-SSFP measurements of myocardial area at risk adds further support that CE-SSFP may be a useful tool in studies investigating interventional, pharmacological and cardioprotective therapies in STEMI. These data and the practicality of adding early contrast enhancement to standard SSFP protocols, makes this a widely applicable method.

#### Limitations

The study was a single center retrospective analysis and thus subject to selection bias that may limit its external validity. The study was a post-hoc analysis from a parent study that was powered for clinical endpoints and so the analysis presented here is open to type 2 error. The results may have been influenced by the post-conditioning protocol and presence of collateral blood supply. The post-conditioning protocol, however, did not affect the overall infarct size. Furthermore, as infarct size was similar, an analysis on the differential predictive capacity for the techniques and infarct size based on therapy could not be performed

The angiographic jeopardy scores are based on anatomical assumptions and the relationships between these and other scoring systems in predicting clinical outcome is unclear.

No semi-quantitative method was used to determine MaR with the CE-SSFP sequence since the signal intensity difference between remote myocardium and MaR is small and makes it difficult to choose a fixed threshold value. Nevertheless, the inter-observer variability was excellent between the image readers. However, a new freely available semi-automatic software for estimating MaR using CE-SSFP has recently been validated and published [19]. This program can be used in all major vendors and can further decrease variability as well as increase the accuracy in estimating MaR for future reperfusion studies.

## Conclusion

Quantification of MaR with contrast-enhanced SSFP imaging following acute STEMI shows high correlation and low bias compared with angiographic scoring and further supports its use as a reliable and practical method to determine myocardial salvage in this patient population.

## Abbreviations

ACE: angiotensin converting enzyme inhibitor
APPROACH: Alberta Provincial Project for Outcome Assessment in Coronary Heart Disease
ARB: angiotensin receptor blocker
BARI: Bypass Angioplasty Revascularization Investigation Myocardial Jeopardy index
CE-SSFP: contrast-enhanced steady-state free precession
CI: confidence interval
CMR: cardiovascular magnetic resonance imaging
IQR: interquartile ranges
LAD: left anterior descending coronary artery
LCx: left circumflex coronary artery
LGE: late gadolinium enhancement
LV: left ventricle
MACCE: major adverse cardiovascular and cerebral events
MaR: myocardium at risk
PCI: percutaneous coronary intervention
RCA: right coronary artery
SD: standard deviation
SPECT: nuclear single photon emission computed tomography
STEMI: ST elevation myocardial infarction
TE: echo time
TIMI: Thrombolysis in Myocardial Infarction
TR: repetition time
